# Study of the Gastrointestinal Heat Retention Syndrome in Children: From Diagnostic Model to Biological Basis

**DOI:** 10.1155/2019/5303869

**Published:** 2019-12-14

**Authors:** Xueyan Ma, Chencheng Mei, Ling Huang, Chen Bai, Jingnan Xu, Yuxiang Wan, Jianhua Zhen, Zhuo Li, Lijun Cui, Shaoyang Liu, Tiegang Liu, He Yu, Xiaohong Gu

**Affiliations:** ^1^Beijing University of Chinese Medicine, Beijing 100029, China; ^2^Beijing University of Chinese Medicine Third Affiliated Hospital, Beijing 100029, China

## Abstract

Gastrointestinal heat retention syndrome (GHRS) refers to a condition that is associated with increased gastrointestinal heat caused by a metabolic block in energy. It is common in children and is closely related to the occurrence and development of recurrent respiratory tract infection, pneumonia, recurrent functional abdominal pain, etc. However, there are no standardized diagnostic criteria to differentiate the GHRS. Therefore, this study is aimed to establish a diagnostic model for children's GHRS and explore the possible biological basis by using systems biology to achieve. Furthermore, Delphi method and the clinical data of Lasso analysis were used to screen out the core symptoms. Nineteen core symptoms of GHRS in children were screened including digestive symptoms such as dry stool, poor appetite, vomiting, and some nervous system symptoms such as night restlessness and irritability. Based on the core symptoms, a GHRS diagnosis model was established using the eXtreme Gradient Boosting (XGBoost) method, and the accuracy of internal verification reached 93.03%. Relevant targets of the core symptoms in the Human Phenotype Ontology (HPO) were retrieved, and target interactions were linked through the Search Tool for the Retrieval of Interacting Genes/Proteins (STRING) database, and core targets were selected after topological analysis using Cytoscape. Relevant biological processes and pathways were analyzed by applying the DAVID and KEGG databases. The enriched biological processes focused on the cell proliferation, differentiation, apoptosis, and mitochondrial metabolism, which were mainly associated with PI3K-AKT, MAPK network pathways, and the Wnt signaling pathway. In conclusion, we established a diagnosis model of GHRS in children based on the core symptoms and provided an objective standard for its clinical diagnosis. And, the Wnt signaling pathway and the estrogen receptor-activated PI3K-AKT and MAPK network pathways may play important roles in the GHRS processing.

## 1. Introduction

Gastrointestinal retention syndrome (GHRS) refers to a condition that is associated with accumulated gastrointestinal heat caused by a metabolic block in energy [1]. GHRS, a state of digestive dysfunction, is associated with diet structure and habits and lack of exercise [2]. Children prefer to intake a high-calorie and high-fat diet for the good taste, which is more likely to cause digestive disorders, for their digestion function is immature and always vulnerable to suffering GHRS in the childhood stage of growth and development.

As a common clinical syndrome from the point of view of Traditional Chinese Medicine (TCM), GHRS has been found to be closely related to the occurrence of many diseases such as recurrent respiratory tract infection and eczema in children [1, 3]. Early intervention on the predisease state of GHRS may prevent some diseases before suffering. Previous experimental studies have shown that GHRS can aggravate inflammatory injury of pneumonia, and pulmonary inflammation can be alleviated after resolving GHRS [4]. Besides, clinical studies prove that GHRS relates to allergic purpura, recurrent functional abdominal pain, etc. [5, 6]. “Different diseases, same treatment,” a unique concept in TCM, refers to the same treatment strategy which is used even in the development of different diseases due to the same pathogenesis. In-depth study of GHRS will be beneficial and meaningful for interpreting the principle of “different diseases, same treatment.”

A GHRS model characterized by a high-calorie diet has been established for the basis of biological research. The experiment found the mechanical barrier damage of the intestinal mucosa, immune dysfunction, trace element imbalance, intestinal microecological disorders, substance/energy metabolism disturbance, etc. [4, 7–11].

Our team has developed the diagnostic scale for GHRS previously [12]. When developing the scale, a self-made standard was used as the diagnostic gold standard. The design principles of self-made standard were not made by experts or based on authoritative literature. Since the symptoms were not well explained and clarified in detail in the previous GHRS diagnostic scale, we found that the subjects could not understand it clearly when they were required to answer the questions.

**Figure 1:**
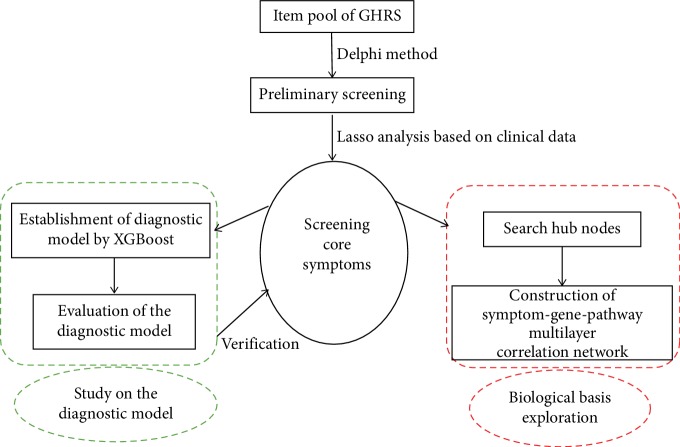
The framework of this study. The core symptoms were obtained by using the Delphi method and Lasso analysis for further study of diagnostic model and systems biology. The good performance of the diagnostic model can prove that the core symptoms are the key to diagnose the GHRS and provide substantial support for the further study of systems biology for GHRS.

This study is aimed to establish a new diagnostic model based on clinical symptoms with reference to the Transparent Reporting of a multivariable prediction model for Individual Prognosis Or Diagnosis (TRIPOD) guidelines [13]. The Delphi method and machine learning based on clinical data were used to establish the model. Meanwhile, based on the core symptoms, we also expect to construct the symptom-gene-pathway network to indicate the biological basis of GHRS by systems biology (Figure 1).

## 2. Materials and Methods

### 2.1. Delphi Method

The Delphi method is a forecasting process framework based on the results of multiple rounds of questionnaires sent to a panel of experts for analysis and judgment on the potential items [14]. In this study, we selected the item pool according to the literature and screened symptoms by two rounds of the Delphi method. We invited 65 experts for the first round online questionnaire investigation. Experts were asked to score each symptom according to the significance in the diagnosis of GHRS using the 5-point Likert scale (4 points indicate very importance, and 0 point indicates unimportance). During questionnaire design, open-ended questions were also designed to fully obtain experts' opinions. After statistical analysis on the first round of experts' questionnaires, some items were added, and the results were fed back to the experts for the second round of questionnaire survey. The effective recovery rate of the expert consultation questionnaire has often been used to reflect positive input from the experts. A 50% recovery rate was the minimum acceptable rate for analysis and reporting, 60% could be considered good, and 70% achieved a very good standard [15]. Cronbach's alpha has often been used to quantify the reliability of a summation of entities. Some scholars believe that Cronbach's alpha of the diagnostic scale should be over 0.90 in clinical trials [16, 17]. Therefore, we used it to evaluate the consistency of expert opinions.

The experts involved in this study were selected from many provinces and cities in China, including TCM pediatrics, TCM diagnostics, and clinical foundation of TCM, and had at least 8 years of clinical experience with senior titles. The main statistical indicators used to analyze the importance of symptoms were mean expert score (i.e., the sum of expert scores/number of participated experts), full-mark rate (number of experts who scored full mark/number of participated experts), and variable coefficient of expert scores (standard deviation/mean score). The symptom would be deleted, if it met one of the conditions as follows: (1) the full mark rate of the symptom lower than the mean full mark rate of each symptom ‐ the standard deviation of full mark rate of each symptom, (2) the mean value of the symptoms lower than the mean value of the mean value of the symptoms ‐ the standard deviation of the mean value of the symptoms and (3) the variable coefficient of the symptom higher than the mean value of variable coefficient of each symptom + the standard deviation of variable coefficient of each symptom.

### 2.2. Clinical Data Preparation

This is a cross-sectional study. The study was conducted during January–December 2018 at the Dongfang Hospital of the Beijing University of Chinese Medicine (BUCM), Dongzhimen Hospital affiliated to BUCM, Guoyi Tang Outpatient Clinic, BUCM, Guandao Central Primary School, Xishiyang Complete Primary School, and Xingjiawu Complete Primary School. Children aged from 3 to 14 years were enrolled. The guardians of the study subjects were informed consent. We excluded patients with serious primary diseases, serious illness, and severe mental disorders in various systems, patients or guardians who did not cooperate with the investigation, and patients with dysphasia.

Information collected included general information of the subjects, the diagnosis on GHRS, and symptoms and signs of the subjects. The main symptoms of GHRS were described in the national standard “Clinic Terminology of Traditional Chinese Medical Diagnosis and Treatment-Syndromes” [18]. But there are still no clear guidelines for the diagnosis of GHRS; nowadays, even GHRS is a common clinical syndrome from the perspective of TCM. It is usually diagnosed by TCM physicians according to their clinical experience in clinic. The title of the clinician refers to the professional technical level, ability, and achievement level of the professional and technical personnel. It can reflect the technical level and working ability of professional technicians. It usually takes the practitioners about 8 years to achieve a vice senior-titled clinician in China after their postgraduation. Therefore, the diagnosis was determined by TCM senior physicians with over 8 years of clinical experience based on the national standard in this study.

The signs and symptoms collected using the Delphi method for initial screening included three parts: observation, pulse taking and palpitation, and interrogation. The first two were collected by TCM professionals after training. The interrogation part was to inquire about the clinical manifestations of the subjects for nearly two weeks, and each symptom was explained and clarified in detail. If there was frequent sweating on the head, factors such as high temperature and spicy diet should be considered and excluded; if the urine was dark, the morning urine should be ruled out. We collected information from the guardians and children. We collected information from their guardians mainly if the children were during 3–7 years old and from the children themselves mainly if they were older than 7 years.

The study was conducted with the human subjects' understanding and consent. It was approved by the Ethics Committee of BUCM (approval number: 2017BZHYLL03110).

### 2.3. Core Symptoms Screened by Lasso Analysis

Item selection based on examining the item-outcome association may include removing items (e.g., based on the *P* value or magnitude of regression coefficients) and shrinking regression coefficients to zero (e.g., by the Lasso method). In this study, the least absolute shrinkage and selection operator (Lasso) method was chosen to select the items [19]. It is applicable for high-dimensional data variable screening [20]. We used R software (version 3.5.2, https://mirrors.tuna.tsinghua.edu.cn/CRAN/, accessed on 30 January 2019) to perform Lasso analysis by using the glmnet package and searched for the average error at the largest lambda within a standard deviation by cross validation, i.e., lambda1se. The screened variables were the core symptoms.

### 2.4. Diagnostic Model Using the eXtreme Gradient Boosting Algorithm

The core symptoms were selected by using Lasso analysis. The eXtreme Gradient boosting (XGBoost) algorithm was used in model establishment. The parameters of XGBoost were optimized for optimal model. After that, the performance of the model was evaluated by the accuracy.

The XGBoost refers to a machine learning element algorithm, which usually sets a weak learner as a base classifier, and then a strong learner is constructed [21, 22]. In this study, we adopted the XGBoost based on a decision tree to achieve extreme gradient improvement by using the XGBoost and the caret packages. The five-fold cross-validation training was used for optimization of parameters. Parameters include nrounds, colsample_bytree, min_child_weight, eta, gamma, subsample, and max_depth. The optimal parameter combination was screened according to the accuracy, and after that, the optimal model was established. The optimal probability threshold for minimizing the error could be found through the information package.

On the test set, we drew the receiver operating characteristic curve (ROC curve) and calculated the area under curve (AUC) [23, 24]. If the AUC >0.9, it indicates a good diagnosis of the model. When analyzing the performance of the model on the test set, we selected five indicators: (1) accuracy, the proportion of correctly diagnosed samples; (2) sensitivity (true positive rate), the ability of judging the sick person to be a patient; (3) omission diagnostic rate (false negative rate), the proportion of misjudging the actual sick person to be a nonpatient; (4) specificity (true negative rate), the ability to correctly judge a disease-free person as a nonpatient; and (5) mistake diagnosis rate (false positive rate), the proportion of misjudging a disease-free person as a patient.

### 2.5. Biological Basis Exploration

Based on the core symptoms in the diagnostic model, we searched relevant genes of symptoms in the Human Phenotype Ontology (HPO) [25, 26]. Then, we imported the genes into the Search Tool for the Retrieval of Interacting Genes/Proteins (STRING) database [27] to find their interactions and import the interactions into Cytoscape 3.7.1 (https://cytoscape.org/, accessed in February 2019) and obtained the topological parameters. Nodes with two times greater than the median degrees were selected as the hub nodes. Their biological processes and pathways were analyzed by using the Database for Annotation, Visualization and Integrated Discovery (DAVID) [28] and the Kyoto Encyclopedia of Genes and Genomes (KEGG) database [29].

## 3. Results

### 3.1. Preliminary Screening of Symptoms Using the Delphi Method

In the first round, 65 experts were invited, and 59 questionnaires (90.77%) were responded. The experts were from 13 provinces and cities such as Beijing, Shanghai, Guangzhou Province, and so on. The average period of working was 27.24 years, and 47 of them (79.66%) had the average years of working ≥20 years. The first round of Cronbach's alpha was 0.974. According to experts' suggestions, items such as night sweating, acid regurgitation, vomiting with smell, and umbilical or gastric tenderness were added. Meanwhile, some items were merged and modified: swift digestion with rapid hungering was modified as abnormal appetite (enormous appetite and poor appetite) and dry mouth and preference for cold drink were merged into thirst with preference for cold drink.

In the second round, 59 questionnaires were delivered, and 54 of them were collected. The effective recovery rate was 91.53%, which achieved a very good standard. The experts were from 13 provinces and cities such as Beijing, Shanghai, Guangzhou Province and so on. The average period of working was 26.63 years, and 45 of them (80.36%) had the average years of working ≥20 years. The second round of Cronbach's alpha was 0.981, indicating a strong consistency of experts' scoring and acceptable results. The indices of each item after the second round Delphi method are shown in Table 1. 10 symptoms including hardened mucus in the nose, acid regurgitation, indigestible food in the stool, pre/postauricular swelling, swelling of the submandibular lymph nodes, cough, expectoration, phlegm wheezing in throat, hyperactivity of the limbs daytime, motor tics, and vocal tics were deleted.

### 3.2. Screening for Core Symptoms by Lasso Analysis

#### 3.2.1. Preparing the Clinical Information Collection Form

According to the Delphi method, some items were deleted. Meanwhile, some items were modified following experts' suggestions, i.e., umbilical or gastric tenderness was divided into two separate items. For pharyngeal symptoms, pharyngeal tonsil swelling was added. The symptoms of being susceptible to respiratory tract infection and oral ulcers were filled out after asking the subjects or guardians about the occurrence in the last year, which were suitable for evaluating the long-term effects of GHRS on patients, but not for diagnosing patient's current syndromes. Therefore, these items were deleted when formulating the clinical information collection form. Finally, the collection form containing 37 symptoms was eventually developed.

#### 3.2.2. Characteristics of Participants

Complete-case analysis was used in this study. A total of 660 effective cases were collected, and divided into the positive GHRS group (*n* = 453) and negative GHRS group (*n* = 207). The general information of all participants is listed in Table 2. There was no significant difference in gender between the two groups, but differed statistically in age. The younger children are more vulnerable to suffering GHRS. The distribution in age of the subjects was in line with the clinical practice.

#### 3.2.3. Screening for Core Symptoms

**Figure 2:**
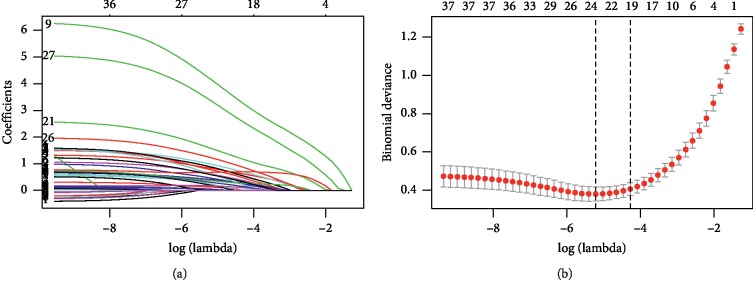
Core symptoms selection using the Lasso regression model. (a) Lasso coefficient profiles of the 37 texture features. A coefficient profile plot was produced against the log *l* sequence. (b) Tuning parameter *l* selection in the LASSO model used 10-fold cross validation via minimum criteria. Binomial deviance was plotted versus log *l*. Dotted vertical lines were drawn at the optimal values by using the minimum criteria, and the 1 standard error of the minimum criteria (the 1-SE criteria). A *l* value of 0.01389958 was chosen (1-SE criteria) according to 10-fold cross validation.

By Lasso analysis, 19 core symptoms were screened from 37 preliminary screening symptoms according to lambda.1se (Figures 2(a) and 2(b)).

### 3.3. Establishing and Evaluating the Diagnostic Model

**Figure 3:**
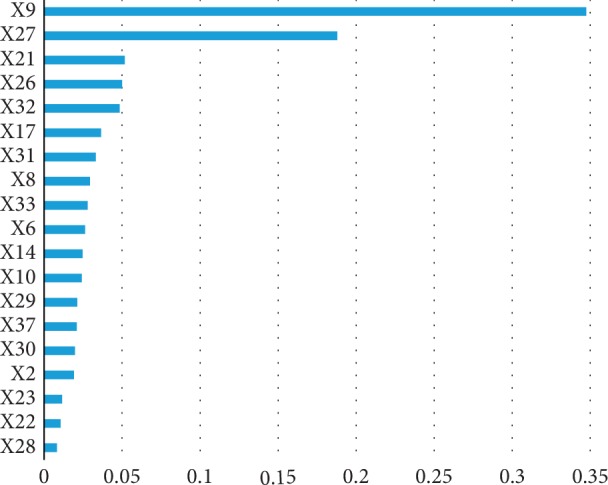
Gain by feature. The gain implies the relative contribution of the corresponding feature to the model calculated by taking each feature's contribution for each tree in the model. It is the most relevant attribute to interpret the relative importance of each feature. The order of each symptoms in significance was thick coating (X9), dry stool (X27), abnormal appetite (X21), reduced frequency of defecation (X26), restlessness at night sleep (X32), halitosis (X17), sweating at night (X31), yellow coating (X8), vexation (X33), red tongue (X6), slippery pulse (X14), feverish feeling in palms and soles (X10), smelly stool (X29), worse after improper diet (X37), yellow urine (X30), red lips (X2), vomiting (X23), belching (X22), and hard defecation (X28).

The model was established by using the XGBoost method. After five-fold cross validation, the highest accuracy was 93.04%. The optimal parameters were as follows: nrounds = 60, min_child_weight = 1, eta = 0.2, gamma = 0.5, colsample_bytree = 1, subsample = 0.55, and max_depth = 4. Based on this, we could obtain the gain value reflecting the significance of the core symptoms (Figure 3). The optimal threshold was determined to be 0.50 by the information package.

**Figure 4:**
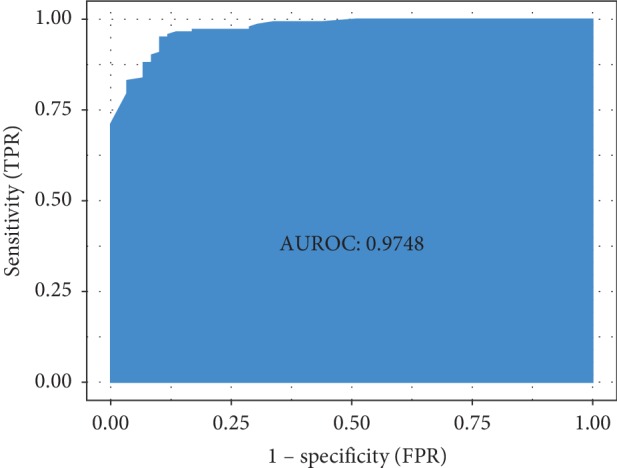
Receiver operating characteristic curve. ROC comprehensively reflects the sensitivity and specificity of the diagnostic model. AUROC indicates the area under ROC curve, which determines the diagnostic value of the model. In this study, the value was 0.9748, suggesting a higher accuracy of the model.

The model performance was analyzed on the test set, and the ROC curve was plotted (AUC = 0.9748; Figure 4). The accuracy rate was 93.03%, which was tantamount to that on the training set. After calculating, the sensitivity, omission diagnostic rate, specificity, and mistake diagnosis rate of the model were 95.07%, 4.93%, 88.14%, and 11.86%, respectively.

### 3.4. Biological Basis Exploration

#### 3.4.1. Search for Hub Nodes

In the HPO, we looked for the relevant HPO entries for 19 core symptoms. Some core symptoms corresponded to multiple HPO entries, such as abnormal appetite corresponding to polyphagia (HP: 0002591), poor appetite (HP: 0004396), and feeding difficulties (HP: 0011968) and anorexia (HP: 0002039). While several core symptoms corresponding to one HPO entry were also existent, such as reduced frequency of defecation, dry stool, and hard defecation, all corresponding to constipation (HP: 0002019) in HPO, symptoms such as fur and pulse images have not been found in the corresponding HPO entries. As shown in Table 3, 14 different HPO entries were found for the 12 core symptoms.

**Figure 5:**
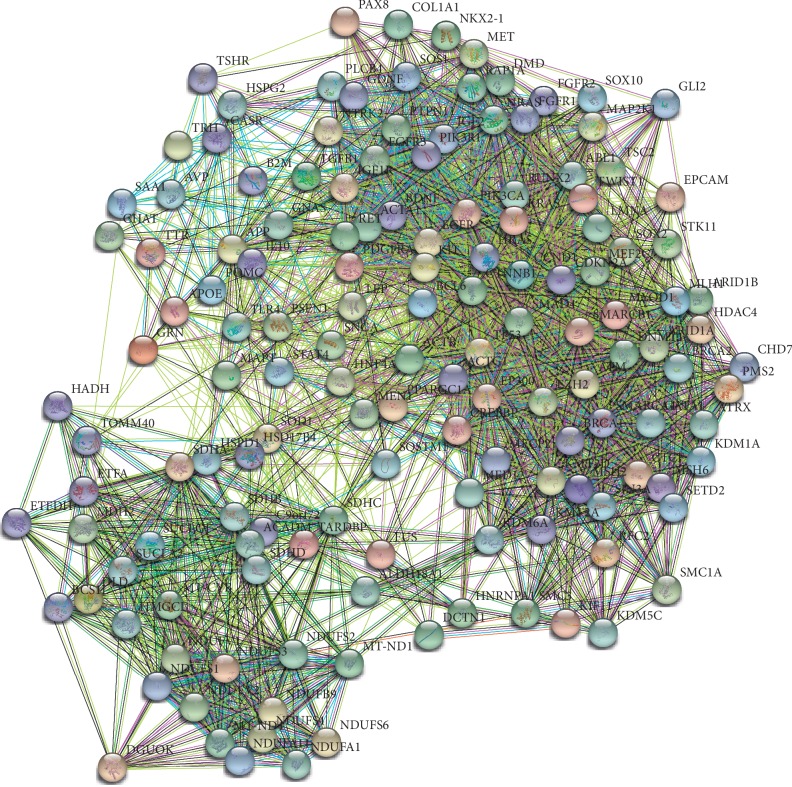
Interactions among core targets.

A total of 905 related nodes were obtained from 14 HPO terms, and 145 hub nodes were screened by degree. The STRING database was used to construct the core target interaction diagram, which produced 145 nodes and 1964 edges. The average node degree was 27.1, the average local clustering coefficient was 0.544, and the enrichment *P* value of the protein-protein interaction was less than 1.0*e* − 16 (Figure 5).

#### 3.4.2. Biological Process and Pathway Enrichment

**Figure 6:**
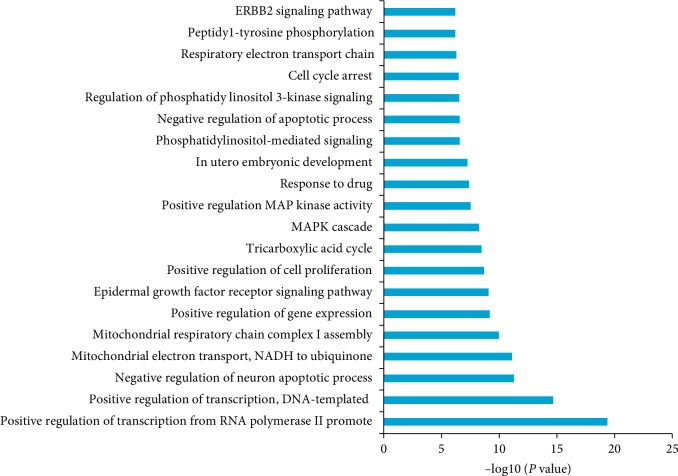
GO biological processes analysis. The sequences were ranked by log10 (*P* value).

The relevant biological processes were analyzed for the core targets by using the Gene Ontology (GO) enrichment, and the first 20 biological processes with *P* < 0.05 were shown in Figure 6, which mainly included two types: cell proliferation, differentiation, and apoptosis and mitochondria-related metabolism.

Biological processes of cell proliferation, differentiation, and apoptosis: (GO: 0045944) positive regulation of transcription from RNA polymerase II promoter, (GO: 0045893) positive regulation of transcription, DNA-templated, (GO: 0043524) negative regulation of neuron apoptotic process, (GO: 0010628) positive regulation of gene expression, (GO: 0007173) epidermal growth factor receptor signaling pathway, (GO: 0008284) positive regulation of cell proliferation, (GO: 0000165) MAPK cascade, (GO: 0043406) positive regulation of MAP kinase activity, (GO: 0048015) phosphatidylinositol-mediated signaling, (GO: 0043066) negative regulation of apoptotic process, (GO: 0014066) regulation of phosphatidylinositol 3-kinase signaling, (GO: 0007050) cell cycle arrest, (GO: 0018108) peptidyl-tyrosine phosphorylation, and (GO: 0038128) ERBB2 signaling pathway.

Biological processes of mitochondria-related metabolism: (GO: 0006120) mitochondrial electron transport, NADH to ubiquinone, (GO: 0032981) mitochondrial respiratory chain complex I assembly, (GO: 0006099) tricarboxylic acid cycle, and (GO: 0006099) respiratory electron transport chain.

Pathway analysis was performed on the hub nodes, and 12 pathways related to cell proliferation, differentiation, and apoptosis were obtained with *P* < 0.05, and 4 pathways related to mitochondrial metabolism were obtained with *P* < 0.05 (Table 4).

#### 3.4.3. Construction of Symptom-Gene-Pathway Multilayer Correlation Network

**Figure 7:**
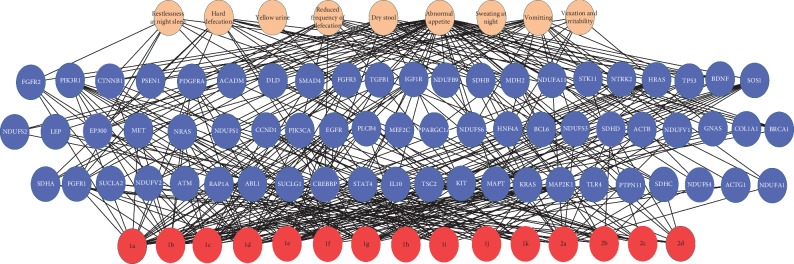
Symptom-gene-pathway multilayer correlation network. The first layer (in pink) represents the core symptoms. The second layer (in blue) represents the core genes. The third layer (in red) represents the enriched pathways, which have been specified in Table 4.

Based on the above results, a symptom-gene-pathway network was constructed with 9 core symptoms, 65 core genes, and 15 pathways (Figure 7).

## 4. Discussion

“Pattern identification and treatment” is a representative feature in TCM. “Syndrome” is a summarization of the nature of the pathogenesis at a certain stage in the occurrence and development of disease [30] and serves as the theoretical basis of “different diseases, same treatment.” An in-depth study of syndromes can profoundly clarify the biological basis of “different diseases, same treatment.” Taking “GHRS” as an example, we intend to explore the research mode of TCM syndromes. We focused on GHRS in two aspects: a clinical diagnosis model of GHRS and a biological network. The diagnostic model based on core symptoms provides objective criteria for the diagnosis of GHRS in clinical practice, which can be used for related disease prevention and treatment. In the biological basis study, we constructed a symptom-gene-pathway network based on the core symptoms, screened out the relevant pathways, and provided ideas for the study of the biological mechanism of GHRS.

When screening for core symptoms, we adopted subjective and objective methods in combination. First, the Delphi method was used to preliminarily screen the symptoms. The key to the Delphi method is the selection of experts. When selecting, we considered regional, authoritative, and professional balance. Then, the clinical data were collected by TCM professionals after training. Each item was explained and clarified in detail to ensure that the information providers (the subjects or their guardians) were able to understand the meaning of each item correctly. The clinical data were analyzed by using Lasso analysis. Lasso analysis can shrink the feature weight to 0 by contracting penalty term, so as to screen the potential variables. Finally, 19 core symptoms were screened from 47 ones.

The core symptoms were picked out by using Lasso analysis based on the clinical data. The core symptoms included digestive symptoms such as reduced frequency of defecation, dry stool, vomiting, abnormal appetite (enormous or poor), and neurological symptoms such as vexation and irritability and night restlessness. Why there were neurological symptoms occurred in GHRS? Previous experimental studies have found that compared with normal mice, serum Ghrelin (a brain-gut peptide) content of the GHRS model mice was significantly reduced [31], and its intestinal microecology was in an unbalanced state [11]. From a modern medical point of view, we speculate that GHRS may be associated with microbiota-gut-brain axis imbalance. The “gut-brain axis” is a network in which the brain and the gastrointestinal tract communicate with each other in two-way signal exchange via nerves, immunity, and endocrine. With the deepening of research, the importance of intestinal microbes in the gut-brain axis has gradually been recognized, thus forming the concept of “microbiota-gut-brain axis” [32]. Some studies have proved that intestinal flora imbalance was related to not only gastrointestinal disorders such as inflammatory bowel disease and irritable bowel syndrome [33, 34], but also mental and neurological diseases such as anxiety, depression, Alzheimer's disease, and Parkinson's disease [35–39].

When establishing the diagnostic model, we chose the XGBoost method suitable for classification and selected the optimal parameters through the cross validation in grid search to optimize the model (AUC = 0.9748). The accuracy of the model reached 93.04% with a satisfactory performance. The diagnostic model transforms the abstract TCM syndrome into a clinical symptom-based diagnostic model, which bases the prevention and treatment of related diseases.

Based on the core symptoms, we constructed a symptom-gene-pathway network after searching related databases. The biological processes and pathways from enriched analysis were mainly involved in cell proliferation, differentiation, apoptosis, and mitochondrial metabolism. Among them, Wnt signaling pathway is very important to pay attention, which plays an important regulatory role in cell differentiation, proliferation, and apoptosis. Some study found that the Wnt signaling pathway was involved in the regulation of mitochondrial homeostasis and mediates mitochondrial stress responses between nerve and intestinal cells [40]. It still remains to be verified by further experiments whether Wnt signaling pathway plays a key role in GHRS.

PI3K-AKT and MAPK are two obvious enrichment pathways, and both of them are involved in cell proliferation, differentiation, and metabolic processes with a cross talk relation [41, 42]. The other multiple pathways enriched are closely related to the PI3-AKT and MAKP pathway networks, such as estrogen receptor (ER) and ErbB mediating PI3-AKT, MAPK pathways [43, 44], FoxO acting as a downstream target of PI3-AKT [45], and ras and rap1 activating the MAPK system [42]. Above all, we focused on the estrogen signaling pathway. Estrogen has an extensive effect on the gastrointestinal and nervous systems and is closely related to gastrointestinal tumors, inflammatory bowel disease, irritable bowel syndrome, Parkinson's disease, schizophrenia, etc. [46–50]. Estrogen may affect the chemical barrier of the intestinal tract through activation of the PI3-AKT pathway by ER [51]. In the brain, ERs interact with MAPK and PI-3K signaling pathways to regulate transcription of target genes [52]. Estrogen and its receptors are involved in the gut-brain axis, and its correlation with GHRS needs further exploration.

There are still some shortcomings in the study. First, the diagnostic model was only internally verified, but was not externally verified. Second, the fur and pulse images played important roles in the diagnostic models, but their corresponding items could not be found in the relevant databases. Finally, further experimental study is required to explore the biological basis of GHRS.

In conclusion, we established a diagnostic model of GHRS, providing an objective standard for the application of this concept as well as a basis for studying the prevention and treatment of related diseases. Besides, we explored and analyzed its potential biological basis based on systems biology. We speculate GHRS is associated with microbiota-gut-brain axis imbalance. Wnt signaling pathway and estrogen signaling pathway may play important roles in GHRS processing. All these provide some new ideas for the biological basic research on GHRS.

## Figures and Tables

**Table 1:** Indices of each item using the delphi method (second round).

Item	Average score	Variable coefficient	Full-mark rate (%)
Red complexion	2.94	0.22	16.67
Aversion to heat	2.61	0.35	14.81
Head sweating	2.78	0.27	16.67
Sweating at night	2.37	0.36	12.96
Feverish feeling in palms and soles	3.48	0.20	57.41
Eye discharge	2.56	0.27	5.56
Epistaxis	2.19	0.33	3.70
Hardened mucus in the nose	1.89	0.41	0.00
Red lips	3.54	0.17	59.26
Gingival swelling	2.78	0.29	18.52
Gingival pain	2.50	0.35	11.11
Halitosis	3.69	0.15	72.22
Hot mouth and nasal breath	3.24	0.17	29.63
Mouth sores	3.07	0.21	24.07
Thirst with preference for cold drink	3.30	0.16	33.33
Abnormal appetite (enormous or poor)	3.15	0.24	35.19
Belching	2.24	0.34	7.41
Acid regurgitation^*∗*^	1.83	0.46	3.70
Vomiting with smell	2.78	0.32	22.22
Distending pain in the abdomen and gastric cavity	2.63	0.31	12.96
Umbilical or gastric tenderness	2.11	0.40	5.56
Smelly flatus	3.54	0.16	57.41
Dry stool	3.50	0.17	55.56
Hard defecation	3.02	0.25	25.93
Reduced frequency of defecation (>once per day)	2.78	0.30	16.67
Indigestible food in the stool	1.87	0.54	7.41
Smelly stool	3.19	0.27	44.44
Susceptible to contract respiratory tract infection	2.65	0.28	11.11
Pharyngeal swelling	2.72	0.33	20.37
Pharyngalgia	2.43	0.35	9.26
Pre/postauricular swelling^*∗*^	1.61	0.47	0.00
Swelling of the submandibular lymph nodes^*∗*^	1.76	0.52	3.70
Cough^*∗*^	1.87	0.48	3.70
Expectoration^*∗*^	1.69	0.56	1.85
Phlegm wheezing in the throat^*∗*^	1.59	0.60	1.85
Vexation and irascibility	2.70	0.35	18.52
Restlessness on night sleep (hyperactivity, odontoprisis, and somniloquy)	3.22	0.21	35.19
Hyperactivity of the limbs daytime^*∗*^	1.94	0.39	1.85
Motor tics or vocal tics^*∗*^	1.44	0.67	0.00
Yellow urine	2.93	0.25	22.22
Red tongue	3.67	0.15	70.37
Red dot on the tongue	2.94	0.31	27.78
Yellow fur	3.65	0.16	70.37
Thick fur	3.54	0.18	61.11
Rapid pulse	3.20	0.20	33.33
Slippery pulse	3.22	0.26	44.44
Worse after improper diet (crapulence or indulgence for greasy foods)	3.35	0.21	48.15
Mean	2.72	0.31	24.59
Standard deviation	0.65	0.13	21.64
Threshold	2.07	0.44	2.94

^*∗*^The items which were deleted.

**Table 2:** Characteristics of the group with GHRS and group without GHRS.

Characteristics	Positive GHRS group	Negative GHRS group	*χ* ^2^	*P* value
Gender, no. (%)			0.157	0.692
Male	231 (50.99)	109 (52.66)		
Female	222 (49.01)	98 (47.34)		
Age range, no. (%),			30.918	≤0.001^*∗*^
3–6years	250 (55.19)	66 (31.88)		
7–14years	203 (44.81)	141 (68.12)		

^*∗*^ *P* < 0.05, which shows there is a significant difference between the two groups.

**Table 3:** Core symptoms and corresponding HPO terms.

Core symptoms	HPO terms
Red lips	Lip telangiectasia (HP: 0000214)
Red tongue	Tongue telangiectasia (HP: 0000227)
Yellow Fur	None
Thick Fur	None
Feverish feeling in palms and soles	None
Slippery pulse	None
Halitosis	Halitosis (HP: 0100812)
Abnormal appetite	Polyphagia (HP: 0002591), poor appetite (HP: 0004396), feeding difficulties (HP: 0011968), and anorexia (HP: 0002039)
Belching	None
Vomiting	Vomiting (HP: 0002013)
Reduced frequency of defecation	Constipation (HP: 0002019)
Dry stool	Constipation (HP: 0002019)
Hard defecation	Constipation (HP: 0002019)
Smelly stool	None
Yellow urine	Abnormal urinary color (HP: 0012086)
Sweating at night	Night sweats (HP: 0030166)
Restlessness at night sleep	Sleep disturbance (HP: 0002360) and bruxism (HP: 0003763)
Vexation and irritability	Restlessness (HP: 0000711) and mania (HP: 0100754)
Worse after improper diet	None

**Table 4:** Pathway pattern underlying core targets.

Symbol	Pathway entry	Pathway description	Count
1a	hsa04151	PI3K-Akt signaling pathway	22
1b	hsa04015	Rap1 signaling pathway	20
1c	hsa04068	FoxO signaling pathway	18
1d	hsa04014	Ras signaling pathway	18
1e	hsa04010	MAPK signaling pathway	17
1f	hsa04510	Focal adhesion	15
1g	hsa04722	Neurotrophin signaling pathway	14
1h	hsa04915	Estrogen signaling pathway	10
1i	hsa04630	Jak-STAT signaling pathway	10
1j	hsa04012	ErbB signaling pathway	9
1k	hsa04310	Wnt signaling pathway	8
2a	hsa00190	Oxidative phosphorylation	14
2b	hsa01200	Carbon metabolism	9
2c	hsa04152	AMPK signaling pathway	9
2d	hsa00020	Citrate cycle	8

## Data Availability

The data used to support the findings of this study are available from the corresponding author upon request.
